# Study on characteristics of fire plume in building facade window under lateral blow

**DOI:** 10.1371/journal.pone.0225120

**Published:** 2019-11-27

**Authors:** Z. P. Bai, Y. F. Li, Y. H. Zhao

**Affiliations:** 1 Beijing Key Laboratory of Green Built Environment and Energy Efficient Technology, Beijing University of Technology, Beijing, China; 2 College of Architecture and Civil Engineering, Beijing University of Technology, Beijing, China; University of New South Wales, AUSTRALIA

## Abstract

The overflow of the flame plume from the window is the main cause of the vertical spread of the fire on the facade of the building. This paper considers the geometry of the window by taking measures to prevent the flame from propagating along the vertical wall. In this paper, a residential building is taken as an example to evaluate the flame plume characteristics through experimental tests and numerical simulations. The objective of the present study is to study the flame plume characteristics under the air blow on the outer window side of the building. The theoretical equations of the flame tilt angle, non-dimensional temperature and non-dimensional velocity are derived. A series of experimental tests were carried out in a reduced-scale building model corresponding to the changes of lateral blow ventilation velocity. Reduced scale numerical simulations were conducted to verify the experiments. Results showed that the flame tilt angle increases with ventilation velocity increases. Meanwhile, the experimental results were compared with the reduced-scale tests and numerical simulations. These showed a good agreement between experimental results and numerical simulations. All these findings provide theoretical basis for building fire prevention outside window.

## 1. Introduction

Flame and smoke diffuse from windows are the main reasons for flame propagation in superstructure [1]. At the same time, windows in a building easily causes flame spread to adjacent buildings, for buildings with large density in city [1]. The danger of flame plume spills out from window is mainly the vertical and horizontal flame propagation. And flame spreads from the super high-rise building window. The flame plume spills out from window was usually rotating flame, which is more dangerous compared with the non-rotating flame. The reason is that the entrainment effect is strengthened by rotating flame. A lot of heat is released, and smoke movement is difficult to predict. Therefore, the flame plume was more dangerous. With building structure changes, flammable walls are widely used with the characteristics of good thermal insulation and easy installation. After flame broke out the window in the building, especially after explosion. Sometimes, the flame is large and the extension length is long. Not only does it destroy the external wall, but also it make flame spread velocity increases [2].

Many previous studies have studied the overflow of flame plume spilled from window. Thomas PH et al. [3] studied the flame spilled from window of burning rooms in a building. The temperature distribution and smoke movement were calculated. With the help of network model, SU S et al. [4] analyzed the the external combustion phenomenon of fire development, which used the temperature and smoke velocity. They concluded that the external combustion could seriously damage ship structure when flame burned a period of time. Oleszkiewicz I [5] conducted the exterior combustion flame by experiments, and they analyzed the radiation effect. It was found that the baffle reduced the heat flux through window. However, little research paid attention to the flame tilt angle, non-dimensional temperature and non-dimensional velocity during the building fire prevention with wind. These three factors are very important for the study of building fire prevention.

As an important part of flame analysis and burning behavior study, research on flame propagation of building facades is the basis of preventing flame diffusion. Thus, it is important to protect personal safety and property safety. It is helpful for fire-extinguishing system with study on flame plume spilled from windows in building facade window under lateral blow. It is an interesting question to understand the law of smoke movement. The characteristics of smoke movement provides useful guidance for building design from the perspective of fire prevention. The study on flame propagation of building facades analyzes the smoke characteristics and laws. Through the combination of building facade, the design prevented flame propagation in the building elevation. It avoids the non-flammability of the excessive fireproofing of the elevation. Effective design of exterior facade of building prevents flame propagation. Therefore, the study focuses on fire prevention in building exterior facade structure type, which is an effective and economical method. It plays a vital role to ensure the people and property safety [6].

Fire is caused by ignition of materials in a building, which continues to grow slowly after a period of time. The air is supplied gradually inadequate. Some of the combustibles liquefy and evaporate to form flammable gases with high temperature in a burning building. The high temperature made the external window crack in the burning room. Then, the flame plume enters the propagation stage. Flame spreads along the three directions of the upper, left and right building. Then, flame plume spills out from the window, accelerating the propagation speed and causing larger losses. This stage is flame propagation process, which plays a vital role in the consequences of fire [6]. The flame propagation velocity is fast in building exterior facade window under lateral wind blow. Flame plume enters the second-floor room from the outer broken window. The combustibles of the upper building, such as curtains, sofas, etc., are ignited and formed a secondary combustion. The flame continues to spread and destroy the outer window of the upper layer building. It leaded to the upper space directly burning. Then, the flame spread more rapidly and does more damage [6].

In recent years, a number of previous studies focus on investigating the burning behavior in the building. A series of full scale and reduced scale experiments were done [7]. It was noticed that the flame spread up through the exterior wall in high-rise residential buildings [7]. Yokoi studied the flame plume characteristics spilled from window on the basis of a large number of experiments with a theoretical model. The temperature and velocity of flame plume varied with window height. And a heat transfer model was established between indoor and outdoor [8]. On the basis of Yokoi’s research, Oleszkiewicz and Lee [9, 10] further revised window fire plume model. Thomas [11] and Quintiere [12,13] proposed the heat transfer model between indoor and outdoor through theoretical analysis. Yamaguchi et al. [14] conducted the dimensionless temperature distribution with window flame plume through small scale model experiment. TANG F et al. [15] proposed the characteristics of free boundary, vertical wall and the flame burning behavior in building facades. A number of theoretical analysis and model experiments were conducted under low pressure and hypoxia environment on plateau.Ma et al. [16] investigated the influence of the opening dimensions of building facade on the flame plume spilled from window. Asimakopoulou et al. [17] described the main externally venting flames thermal characteristics in a medium- and a large-scale compartment-façade fire test. Miao L et al. [18] conducted experiments to examine the fast growth of window ejecting flame height influenced by the vertical wall via partially blocking air entrainment on the by-wall side. Ren F et al. [19] investigated experimentally the lateral temperature profiles of window-ejected facade fire plume from compartment with external ambient wind normal to the facade. Hu L et al. [20] investigated facade flame height and horizontal extending distance from opening of compartment re with external sideward wind. Hu L et al. [21] proposed a physics-based non-dimensional correlation, providing basic understanding of the fire dynamics inside a compartment with an opening under external sideward wind. However, the theoretical equations of the flame tilt angle, non-dimensional temperature and non-dimensional velocity have not been deduced in detail.

For fire safety, it is important to understand the flame propagation under the lateral blow. The movement way of flame affects the upper building. To address the problem, the fire flame height and the temperature are investigated in different experimental tests. For the latter point, the flame tilt angle are investigated as an important factor for affecting flame propagation. Livkiss K [22] studied the flame heights in façade system ventilation cavities. Konno Y [23] studied the downward flame propagation over electric wire under various oxygen concentrations. Lam C S [24] conducted experiments to study the temperature field when wind-blown pool fire. Faveri [25] carried out experimental tests to provide exact correlations for predicting flame length of fire source in a ventilation tunnel. Hu L [26] studied the flame soot and radiation emission coupling with complex flow turbulence scales due to the interaction of buoyancy with wind for wind-blown pool fires. However, few studies have been conducted on the flame tilt angle corresponding to the changes of lateral blow ventilation velocity.

The aim of this study is to provide additional research to the previously performed studies [7]. The experimental model was built for studying on characteristics of fire plume in building facade window under lateral blow. A program was performed with experimental setup. The experiment used methanol and N-heptane as fuels. The characteristics of flame plume were studied in building facade window under lateral blow. In this paper, the burner was placed at the middle of the combustion chamber. The experimental configuration provided data for analyzing the heat transfer and flame propagation over the surface outside the fire room. The flame tilt angle, the non-dimensional temperature and the non-dimensional velocity are studied by the wind laterally blowing towards the building. Reduced-scale tests and numerical simulations are conducted for demonstrating the theoretical derivation.

## 2. Method

### 2.1 Theoretical analysis

#### 2.1.1 The flame tilt angle

**Fig 1:**
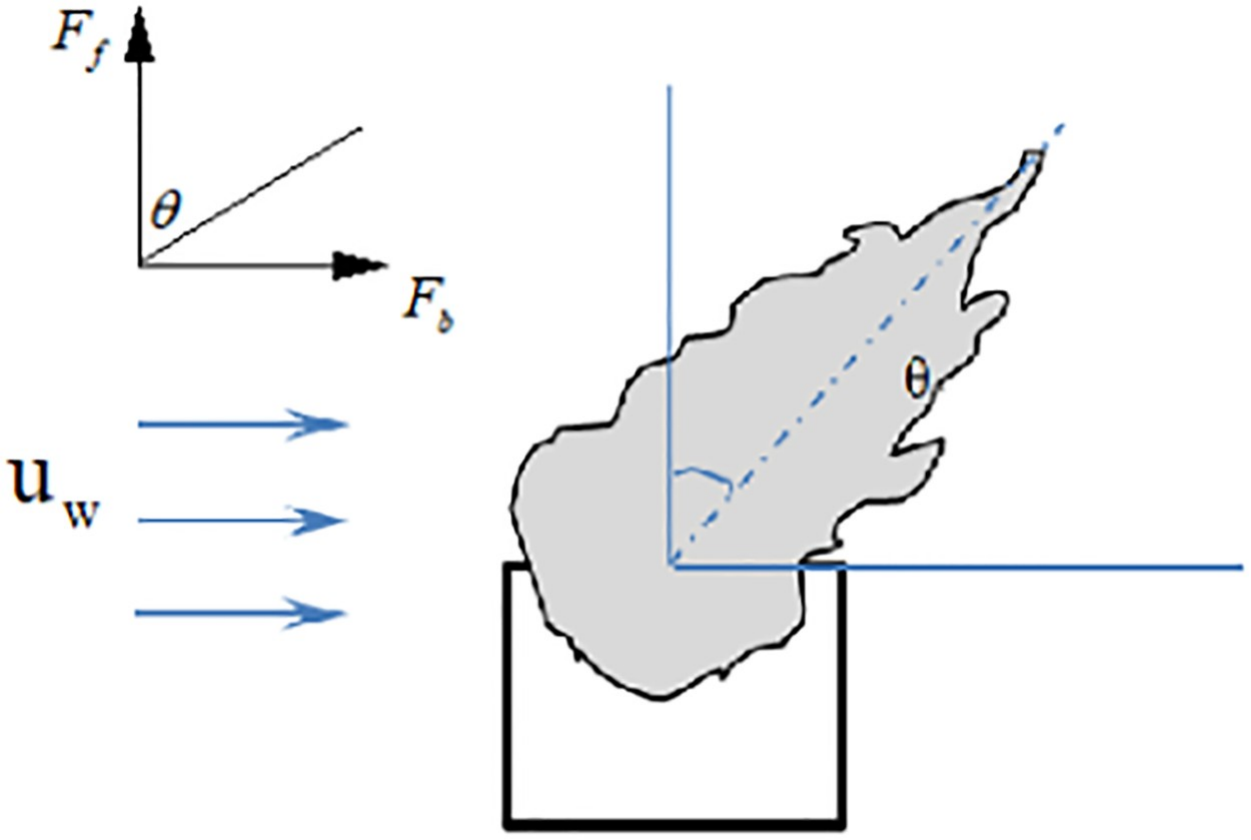
Window plume flow under side blow.

The fire plume spills from window would be deflected under the wind effect of lateral blow, as shown in Fig 1. The flame tilt angle θ is mainly affected by the thermal buoyancy *F*_*f*_ in the vertical direction and the volume force *F*_*b*_ produced by the wind speed in the horizontal direction [27]. The flame tilt angle θ could be expressed by Eq (1).

**Fig 2:**
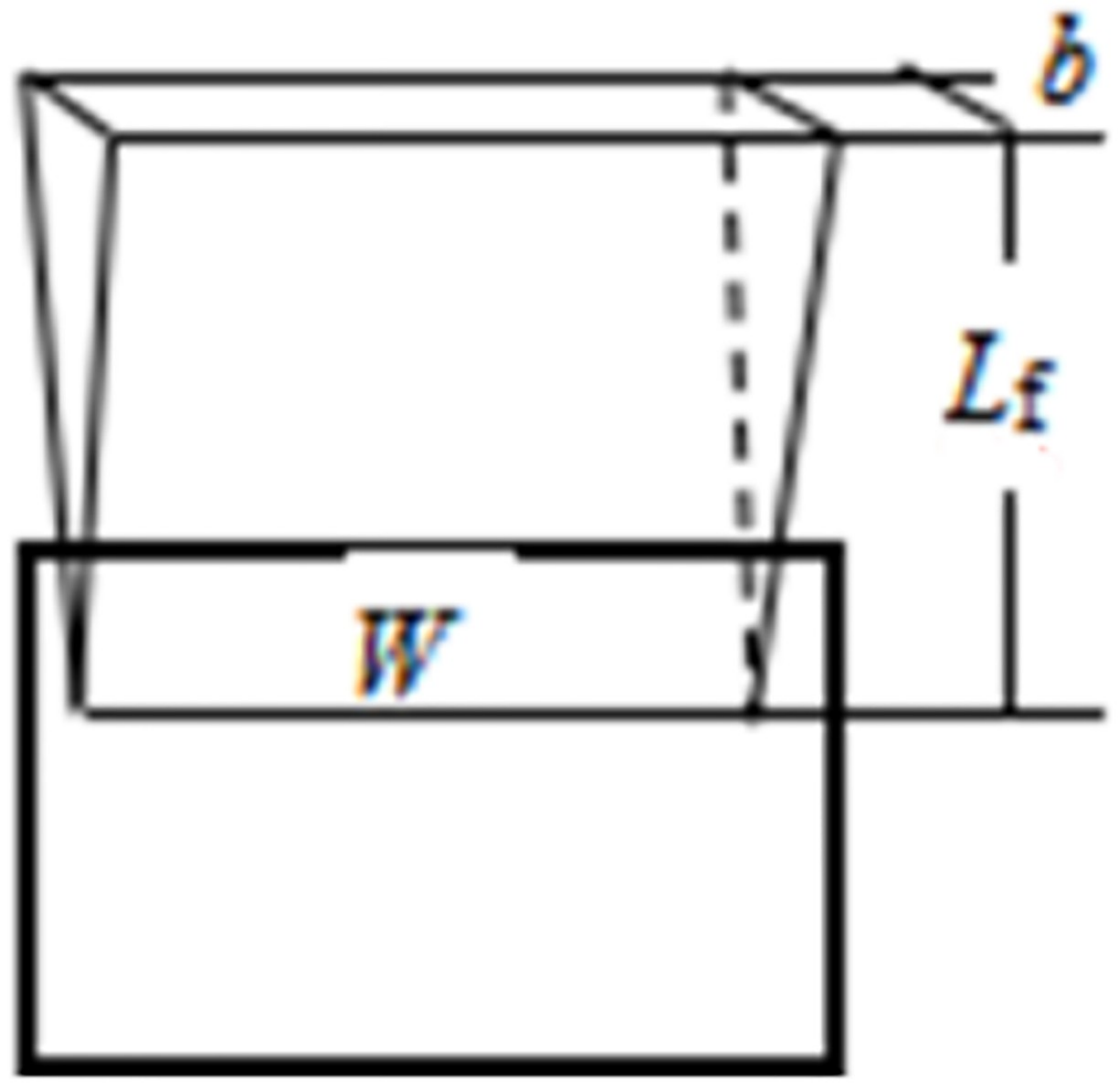
Window plume structure.

It was assumed that the window flame plume height is *L*_*f*_, the flame could be generally regarded as linear spilled flame length W. And the spilled flame is approximated as an inverted vertebral body, as shown in Fig 2. Where, the width of spilled flame from window is b at the height of Z.

$$\text{tan}\;\theta =\frac{F_{b}}{F_{f}}$$ (1)

The heat floating lift in the vertical direction is shown in Eq (2).

$$F_{f}\propto g\left(\rho_{a}-\rho_{g}\right)L_{f}bW/3$$ (2)

External wind force *F*_*b*_ is shown in Eq (3).

$${\text{F}}_{\text{b}}\propto \frac{\rho_{\text{a}}{\text{u}}_{\text{w}}^{2}}{2}\cdot \frac{{\text{L}}_{\text{f}}\text{b}}{2}$$ (3)

$$\rho_{0}T_{0}=\rho_{a}T_{a}$$ (4)

It is known from the previous study that the flame and smoke spilled from window of the fire room can be regarded as a virtual square fire source [27]. And the flame tilt angle θ under the influence of lateral blow is mainly influenced by the vertical force of the hot floating and the inertia force produced by the horizontal direction under lateral blow. It is assumed that the height of the neutral surface is H_s_. Thereafter, the flame tilt angle is shown in Eq (5) [27].

$$\text{tan}\;\theta =\frac{F_{b}}{F_{f}}\propto \frac{3\rho_{a}u_{\text{w}}^{2}}{4\left(\rho_{a}-\rho_{0}\right)gW}$$ (5)

where, the wind velocity is *u*_w_, and g is the acceleration of gravity. *L*_*f*_ is the flame height, which is defined as flame spilled from window. Then, the flame tilt angle is shown in Eq (6).

$$\text{tan}\;\theta \propto \frac{C_{1}T_{0}u_{\text{w}}^{2}}{\left(T_{0}-T_{a}\right)gW}$$ (6)

$$Fr=\frac{u_{\text{w}}^{2}}{gL_{\text{f}}}$$ (7)

Fr is shown as in Eq (7), which is revised. Then, the flame tilt angle is shown in Eq (8).

$$\text{tan}\;\theta =\frac{C_{1}T_{0}}{\left(T_{0}-T_{a}\right)}\frac{L_{\text{f}}}{W}\frac{u_{\text{w}}^{2}}{gL_{\text{f}}}{=\text{C}}_{2}{\text{Fr}}^{\text{m}}$$ (8)

where, the parameter ${\text{C}}_{2}=\frac{C_{1}T_{0}}{\left(T_{0}-T_{a}\right)}\frac{L_{\text{f}}}{W}$.

#### 2.1.2 Non-dimensional temperature

Dimensionless fire source power Q* is shown in Eq (9) [28].

$$Q^{*}=\frac{Q}{\rho_{a}C_{p}T_{a}g^{1/2}{\text{D}}^{5/2}}$$ (9)

Δ*T* is the average temperature change value flame spilled from the window of fire room. The non-dimensional temperature [14], defined by

$$\Theta =(\Delta {\text{T}}_{0}{\text{r}}_{0}^{5/3})/{\left(\frac{T_{a}Q_{D}^{2}}{C_{p}^{2}\rho^{2}g}\right)}^{1/3}$$ (10)

where, ΔT_0_ is the temperature rise at arbitrary position along the fire plume axis, r_0_ is the equivalent radius of the window jet, *T*_*a*_ is the ambient temperature, *Q*_*D*_ is the heat ejected the room along with the window jet, *C*_*p*_ is the specific heat of air, *ρ* is the gas density at arbitrary position along the fire plume axis. The non-dimensional temperature could be expressed by Eq (11).

$$\Theta =(\frac{\Delta {\text{T}}_{0}/{\text{T}}_{\text{a}}}{Q_{D({\text{r}}_{0})}^{*}})/{\left(\frac{T_{0}}{T_{a}}\right)}^{2/3}$$ (11)

Mass flow of window flame plume *m*_*D*_ is defined as:

$$m_{D}=\rho_{\text{g}}L_{\text{f}}^{}bW/3$$ (12)

Heat release rate of window flame plume *Q*_*D*_ is defined as:

$${\text{Q}}_{D}{=\text{C}}_{\text{p}}m_{D}\Delta T$$ (13)

$${\text{Q}}_{D}{=\text{C}}_{\text{p}}\Delta T\rho_{\text{g}}L_{\text{f}}^{}bW/3$$ (14)

T_0_ is the temperature at arbitrary position along the flame plume axis, T_0_ = ΔT_0_ + T_a_.

$Q_{D({\text{r}}_{0})}^{*}$ is the non-dimensional heat flow rate of window jet [14], define as

$$Q_{D({\text{r}}_{0})}^{*}=\frac{{\text{Q}}_{\text{D}}}{{\text{C}}_{\text{p}}\rho_{a}T_{a}\sqrt{g}r_{0}^{5/2}}$$ (15)

$$Q_{D({\text{r}}_{0})}^{*}=\frac{\Delta TL_{\text{f}}^{}bW/3}{T_{\text{g}}\sqrt{g}r_{0}^{5/2}}$$ (16)

$$\Theta =\frac{3\sqrt{g}r_{0}^{5/2}}{L_{\text{f}}^{}bW}/{\left(\frac{T_{0}}{T_{a}}\right)}^{1/3}$$ (17)

$$Q_{D(\text{H})}^{*}=\frac{{\text{Q}}_{\text{D}}}{{\text{C}}_{\text{p}}\rho_{a}T_{a}\sqrt{g}{\text{H}}_{}^{5/2}}$$ (18)

$$Q_{}^{’*}=\frac{{\text{Q}}^{\prime }}{{\text{C}}_{\text{p}}\rho_{a}T_{a}\sqrt{g}{\text{W}}_{}^{5/2}}$$ (19)

Q′ is the heat release rate per unit length at line heat source. W is the width of the line heat source. H is the length. It is assumed that W = H. Q′* is the non-dimensional heat release rate of line heat source. Q′ = Q_D_/B, where B is window width. *ρ*_a_ is the ambient air density. Then, the non-dimensional heat release rate of line heat source ${\text{Q}}_{D(H)}^{\prime *}$ could be shown in Eqs (20)–(21). L_f_ is the height of the window jet.

$$Q_{D(\text{H})}^{\prime *}=\frac{{\text{Q}}_{\text{D}}}{{\text{C}}_{\text{p}}\rho_{a}T_{a}\sqrt{g}{\text{BH}}_{}^{3/2}}$$ (20)

$$Q_{D(\text{H})}^{\prime *}=\frac{{\text{Q}}_{\text{D}}}{{\text{C}}_{\text{p}}\rho_{a}T_{a}\sqrt{g}{\text{BL}}_{\text{f}}^{3/2}}$$ (21)

$$\text{n=}\frac{\text{W}}{{\text{z-L}}_{\text{f}}}$$ (22)

$$z^{*}=\frac{z}{\text{H}}$$ (23)

$$\Theta (0,0,z^{*})=\alpha z^{*-3/5}$$ (24)

$$\alpha =\{\begin{array}{ll} 0.45{\text{n}}^{3/2}, & \left(\text{n}\leq 3.3\right)\\ 2.7, & \left(\text{n}>3.3\right) \end{array}$$ (25)

$$\Theta (0,0,z^{*})=0.66\alpha$$ (26)

where, n is the aspect ratio of the window jet; z* is the non-dimensionless height; 1/*α* is the inclination of the straight line. Θ(0, 0, z*) is the non-dimensionless height z*; They are shown in Eqs (22)–(26) [14].

Window factor is defined as

$$\eta =\text{A}\sqrt{\text{H}}$$ (27)

where, *η* is the window factor; A is the window area, ㎡; H is the window height, m.

The equivalent of a virtual fire under the window plume. $Q_{D(\text{H})}^{\prime *}$ is the ejection height of window flame plume L_f_, which is obtained in Eq (28)

$$Q_{D(\text{H})}^{\prime *}{=\text{Q}}^{*}-1500\text{A}\sqrt{\text{H}}$$ (28)

#### 2.1.3 Non-dimensional velocity

Under the effect of wind lateral blow outside window, the upward flow of the flame plume spills from window lean downward. The high temperature zone of flame and smoke also moves downward, causing flame propagation over the downstream. When smoke emits from the window of the fire room, the flame tilt angle is θ under lateral blow effect for the building. The flame tilt angle is mainly influenced by the wind speed and the heat release rate (HRR) of fire source. The main factors affected the flame tilt θ are the wind speed and the HRR in fire source location. If the flame plume spills out from window was important along the lateral blowing outside the building.

The dimensionless wind speed, V′is defined as:

$${\text{V}}^{\prime }{=\text{u}}_{\text{w}}/(\frac{gQ_{\text{D}}}{\text{W}\rho_{\text{a}}{\text{c}}_{\text{p}}{\text{T}}_{\text{a}}})$$ (29)

The non-dimensional heat release rate of line heat source, *Q*′* is defined as Eq (19). Then, $Q_{D(\text{H})}^{\prime *}$ is the ejection height of window jet L_f_, which is shown in Eq (21).

Eqs (21) and (29) are solved to obtain Eq (30)

$${\text{V}}^{\prime }{=\text{u}}_{\text{w}}^{2}\frac{{\text{Fr}}^{3/2}}{Q_{D(\text{H})}^{\prime *}}$$ (30)

Through theoretical analysis, the Eq (8) could get the flame tilt angle θ. The Eq (26) obtains the non-dimensional temperature Θ. The Eq (30) is used to calculate the non-dimensional velocity V′. Through these theoretical analyses, the characteristics of side-blown air could be understood in detail.

### 2.2 Reduced-scale experimental tests

The approach of physical model was established in previous studies in the field of fire technology. A series of experiments were conducted for fire prevention with a 1:8 reduced scale building. The model was 2.8m long, 0.6m wide and 1.8m high. The whole model was made of cast iron, with a total of three layers. There were two different rooms in each layer. The bottom was set to the combustion chamber and the rest was the retaining wall, which was used to represent the external facade of the building. Small combustion chamber was 0.5m long, 0.5m wide and 0.4m high. Large combustion chamber was 1.0m long, 0.5m wide and 0.4m tall. The wall of the combustion chamber was covered with fireproof and insulating materials. The combustion chamber had adjustable windows, which could change the window area by loading or unloading fireproofing boards. In addition, the bottom combustion chamber and the two stories partition could be moved, in order to ensure the height of the combustion chamber was adjustable. The rear part of the experimental platform was equipped with a sliding door to make up combustion air. The window were 0.15m wide and 0.4m high in this experiment. Fuel plate was 0.1m long, 0.1m wide and 0.1m tall.

**Fig 3:**
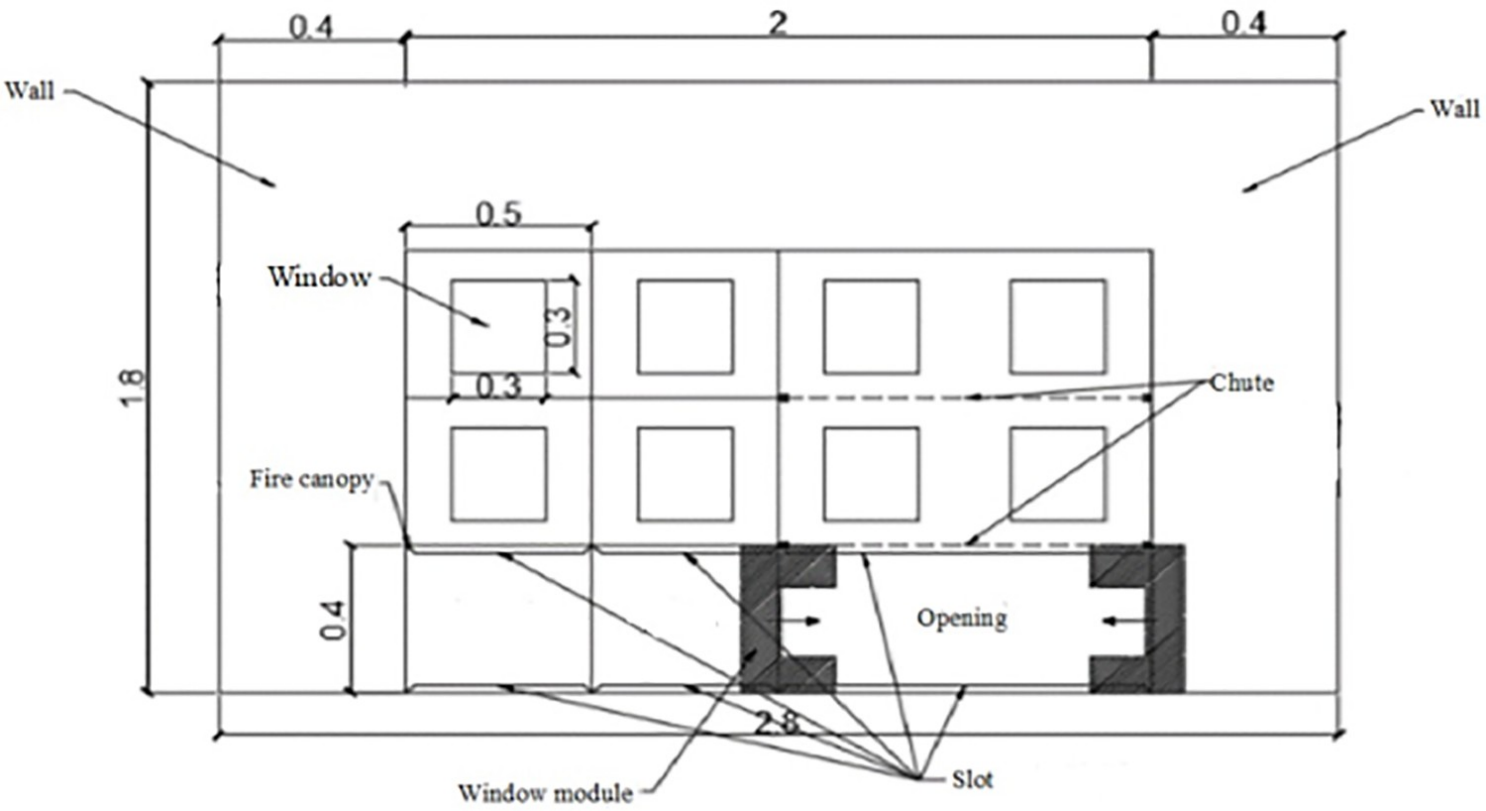
Test bench body design.

**Fig 4:**
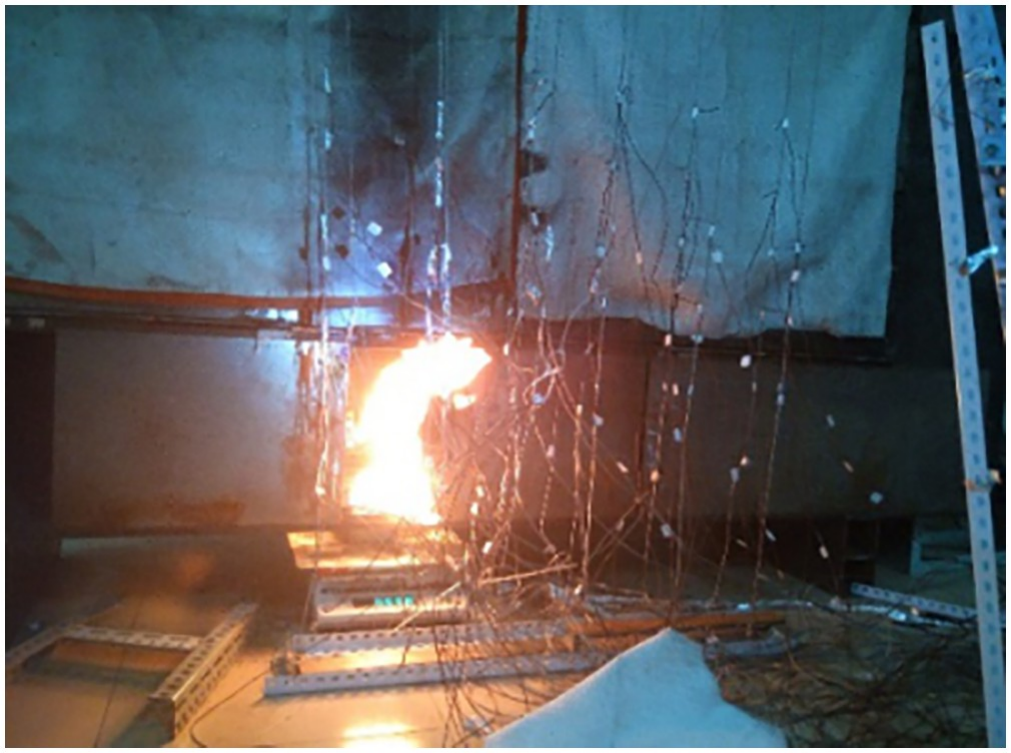
Diagram of the experimental bench.

The schematic of the experimental apparatus was shown in Figs 3 and 4. Methanol and N-heptane were used as fuels. The ambient temperatures is 10°C. Temperature were measured with K-type thermocouples of 1 mm diameter. Type K thermocouples were used with measurement uncertainty less than 3%. Two series thermocouples T1~T10 are placed at the center of the ceiling of the tunnel. One series of thermocouples T1~T5 are placed at a space of 0.05m far away from the building. The other one series of thermocouples T6~T10 are placed at a space of 0.1 m far away from the building. There are 5 thermocouples in each thermocouple tree. And the spacing between each thermocouple tree is 0.2m. The height measurement 5 points were 0.9 m, 0.76 m, 0.62 m, 0.48 m, 0.34 m and 0.20 m, respectively. The thermocouples are arranged from the first thermocouple to bottom at an equal spacing of 0.14 m.

When fire occurred, the flame tilt angle was affected by the external wind speed in the window and the exterior wall. When the external wind was perpendicular to the building, the magnitude of the wind speed could affect the flame tilt angle between the window flame plume and the exterior wall. However, the wind did not make the flame propagation upward along the vertical direction of the building. When a building was blown parallel and sideways, the flame plume moved upward along the vertical direction of the building. In this case, the wind speed did not change the flame tilt angle between the extrusion flame from the window and the exterior wall [29]. Wind speed increased with the building height increased. However, when the building height increased to a certain value, the building height continued to increase, the wind speed did not change with the building height. At the same time, the wind speed was also related to the roughness of the ground. The roughness was increased, when the wind speed was increased [30]. The wind speeds were 0 m/s, 0.6 m/s, 1.3 m/s and 2 m/s, respectively. The experimental conditions as shown in Table 1. The temperature was obtained by experimental tests. The non-dimensional temperature was calculated.

**Table 1:** Experiment conditions.

Case	Fuel	Maximum HRR (kW)	Wind speed (m/s)
1	Methanol	6.81	0.0
2		9.08	0.6
3		9.08	1.3
4		17.84	2.0
5	N-heptane	6.81	0.0
6		9.08	0.6
7		9.08	1.3
8		17.84	2.0

### 2.3 Numerical experiments

**Fig 5:**
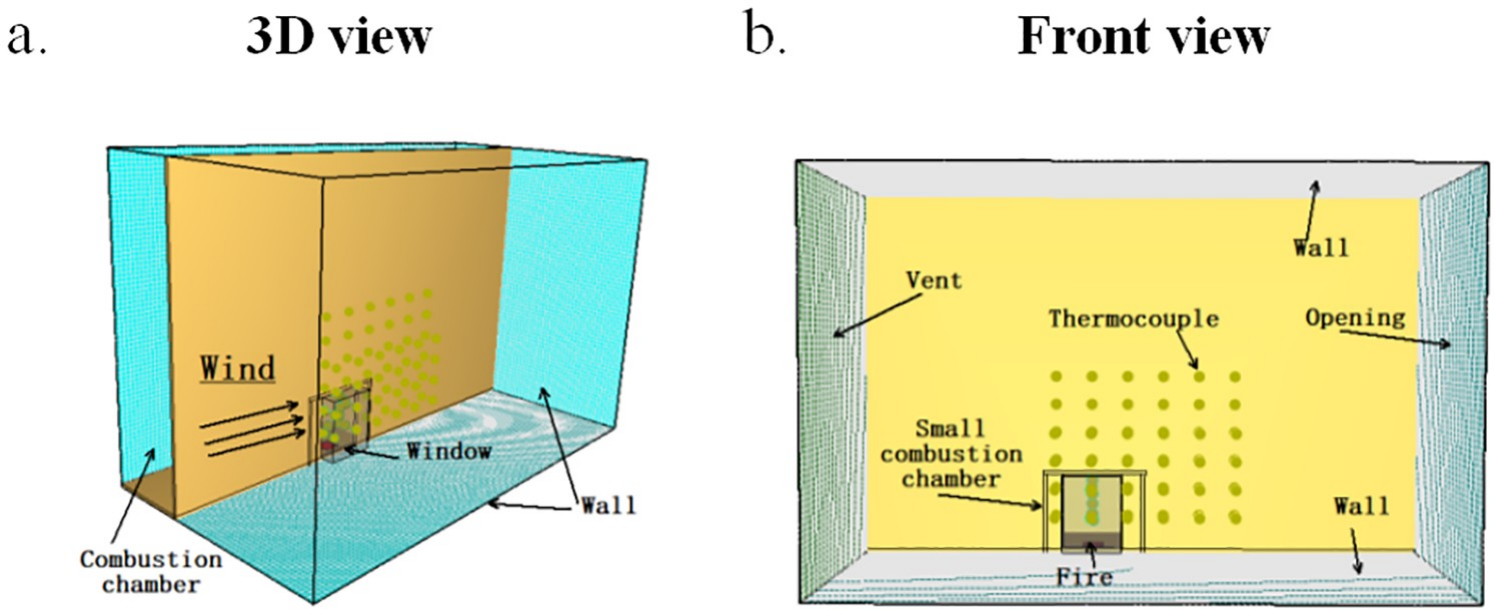
Schematic diagram of the numerical model. (a) 3D view. (b) Front view.

In order to compare and verify the experimental results, the FDS (Fire Dynamics Simulator, version 2017) was used to conduct the experimental tests and reduced scale numerical simulations. A previous study revealed there was good agreement between reduced-scale experiments and simulations for a building configuration [7]. The schematic diagram of numerical model was shown in Fig 5. The size of numerical model, the location of fire source, the characteristics of fire source and the setup of the environment parameters were consistent with the experimental tests, and the location of test points were the same as that of the experiments.

The numerical model was 2.8m long, 0.6m wide and 1.8m high. Small combustion chamber was 0.5m long, 0.5m wide and 0.4m high. Large combustion chamber was 1.0m long, 0.5m wide and 0.4m tall. The window was 0.15m wide and 0.4m high. The ambient temperatures was 10°C. The thermocouples positions were the same as the reduced-scale experiments. The wall is adiabatic. The chamber surface material including walls, ceilings and floors was set as “CONCRETE” specified in the FDS. In the study, the thermal properties of concrete include thermal conductivity of 1.8 W/(m·K), density of 2280 kg/m3 and specific heat of 1.04 kJ/(kg·K). The wind came from left side in Fig 5(a). The left side except chamber was set as “VENT” and the wind speeds were 0 m/s, 0.6 m/s, 1.3 m/s and 2 m/s, respectively. The right side was set as “OPEN”.

The N-heptane was selected as fuel of fire source. The HRRs were 6.81kW, 9.08kW, 17.84kW, respectively. The numerical simulation cases were shown in Table 2. The purpose of numerical simulations were to get the information of temperature distribution, further calculation of the flame tilt angle.

**Table 2:** Numerical simulation conditions.

Case	Fuel	Maximum HRR (kW)	Wind speed (m/s)
9	N-heptane	6.81	0
10			0.6
11			1.3
12			2.0
13		9.08	0
14			0.6
15			1.3
16			2.0
17		9.08	0
18			0.6
19			1.3
20			2.0
21		17.84	0
22			0.6
23			1.3
24			2.0

**Fig 6:**
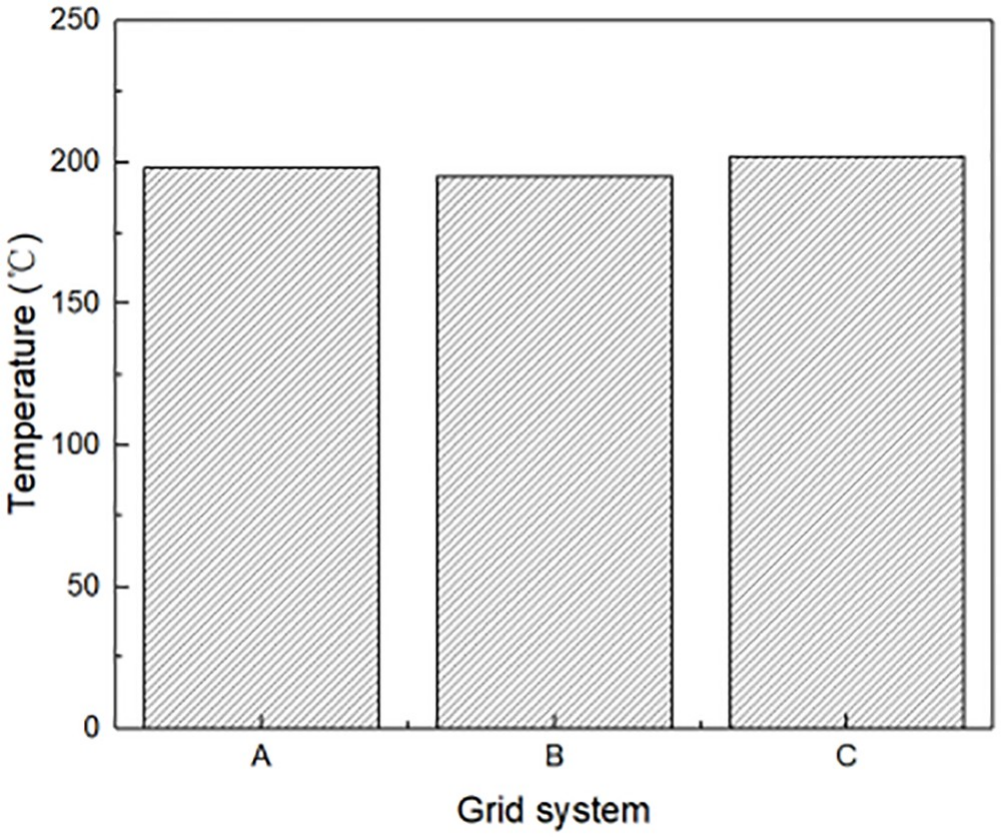
Temperature at window location for three mesh sizes.

The FDS user’s guide [28] suggests a non-dimensional expression of D*/*δx* for assessing a mesh resolution with $D^{*}={\left(\frac{Q}{\rho c_{p}T_{a}\sqrt{g}}\right)}^{2/5}$. The recommended value of D*/*δx* in the range of 4–16. Therefore, for the simulated HRR 6.81 kW~17.84 kW, the grid size is calculated between 0.01 m and 0.2 m. The numerical simulation domain is divided into 2.8 m×0.60 m×1.80 m along the length, width and height direction. In order to verify the feasibility of the existing grid size, grid-independent calculations were performed using three continuous grids. The grid system A is 0.02 m×0.02 m×0.02 m along the length, width and height direction. The grid system B is 0.01 m×0.01 m×0.02 m along the length, width and height direction. The grid system C is 0.04 m×0.04 m×0.04 m along the length, width and height direction. Taking fire scenario with HRR of 9.08 kW as an example, for the wind speed is 1.3 m/s, the temperature at window location under different mesh sizes were calculated. Fig 6 presents the temperature at window location for three mesh sizes. It can be seen that there is little difference between the predicted results by grid system A and grid system B. For the balance of expected precision and time cost, the grid system A is adopted for simulation cases, which is similar with work by Li et al. [7], in the building fire simulation.

## 3. Results and discussion

### 3.1 Temperatures at window and fire source location

**Fig 7:**
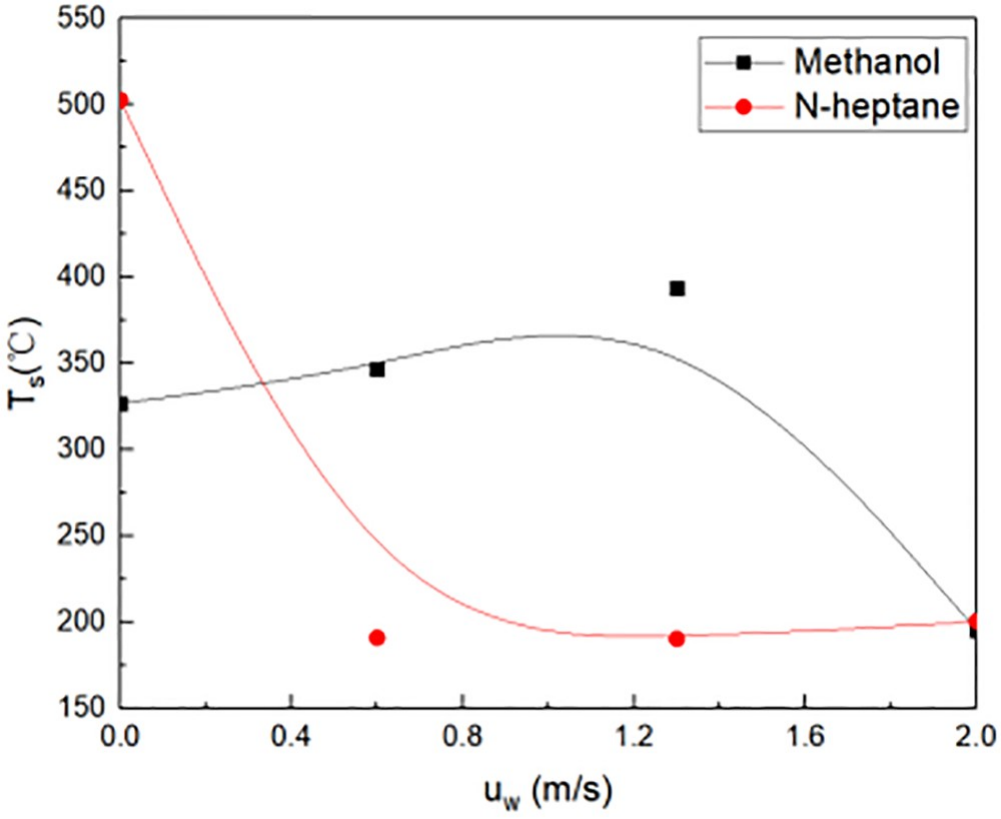
Temperature at fire source in experimental tests.

On one hand, the methanol was used as fire source in experiments. The experimental results were described in Fig 7. As shown in Fig 7, the temperature T_s_ was 300 °C at the fire source without wind outside building. With the wind speed increases 0.6m/s, the temperature was 200 °C at the fire source location. The reason was that wind speed increased, heat was diffused from the combustion room. When the wind speed was greater than 0.6m/s, the basic temperature was 200 °C at the fire source location. The reason for this phenomenon was that the wind speed brought into enough fresh air to make the fuel burn more violently.

On the other hand, the N-heptane fuel was used as fire source in experiments. As shown in Fig 7, the temperature T_s_ was highest at the fire source location. Without wind speed outside window, the value was 500 °C at the fire source location. When the wind speed was 0.6m/s, the temperature was 160 °C, which was lowest at the fire source location. When the wind speed was larger than 0.6m/s, the temperature T_s_ gradually increased at the fire source location.

**Fig 8:**
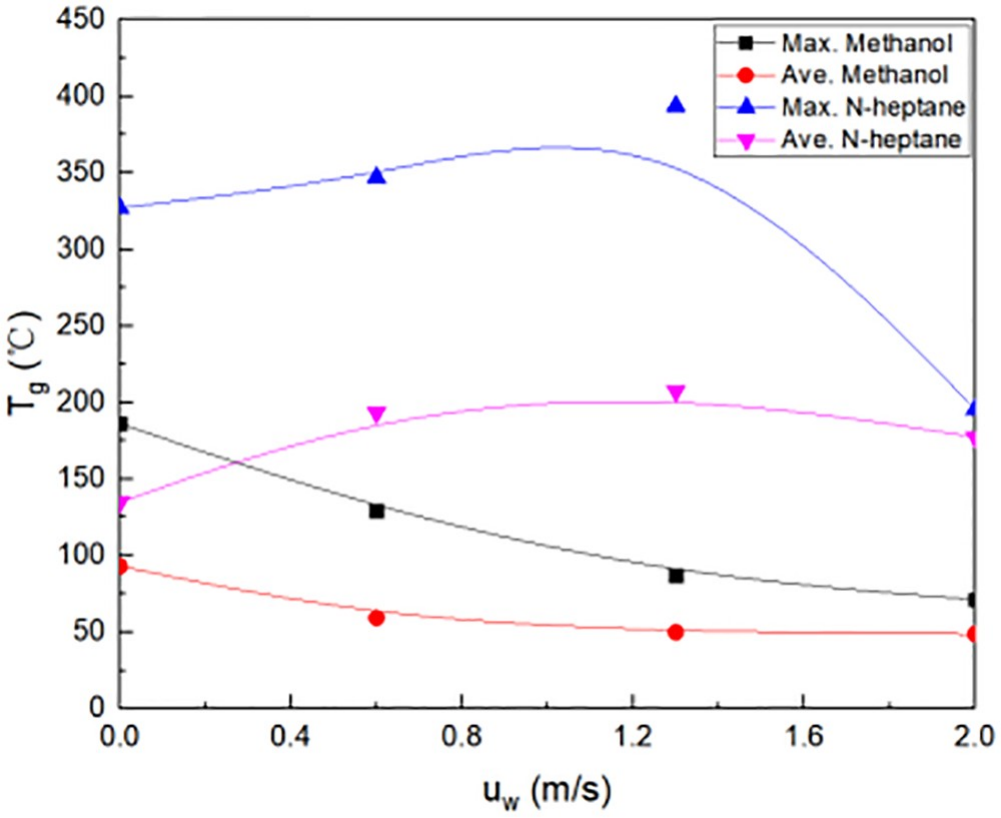
Temperature at window location in experimental tests.

The effect of fuel on the temperature at window location was also investigated. First, the methanol was used as fire source in experiments. As shown in Fig 8, temperature T_g_ was measured at window location in experiment. At this time, the maximum temperature was 170 °Cat the window, while the average temperature was less than 100 °C at the window. When the wind speed outside window changed from 0 m/s to 2.0 m/s, the average temperature decreased gradually at the window location, and the maximum temperature at the window also decreased gradually. When the wind speed outside window exceeded 1.3 m/s, the average temperature kept 50 °C, and the maximum temperature maintained 60 °C at the window location. The reason was that the increase in heat taken from the window as the wind speed increased.

Second, the N-heptane fuel was used as fire source. Without ventilation, the maximum temperature was 320 °C, while the average temperature was about 130°C at the window location. As shown in Fig 8, the maximum temperature first increased, and then decreased at the window location. The maximum temperature was nearly 400°C at the window when the lateral blow wind speed was 1.3m/s. The average temperature increased at the window, when the lateral blow wind speed ranged from 0m/s to 1.3m/s. And then it decreased when the wind speed outside window was more than 1.3m/s. The average temperature was nearly 200°C, which was maximum at the window, when the lateral blow wind speed was 1.3m/s.

### 3.2 Non-dimensional temperature Θ at opening

**Fig 9:**
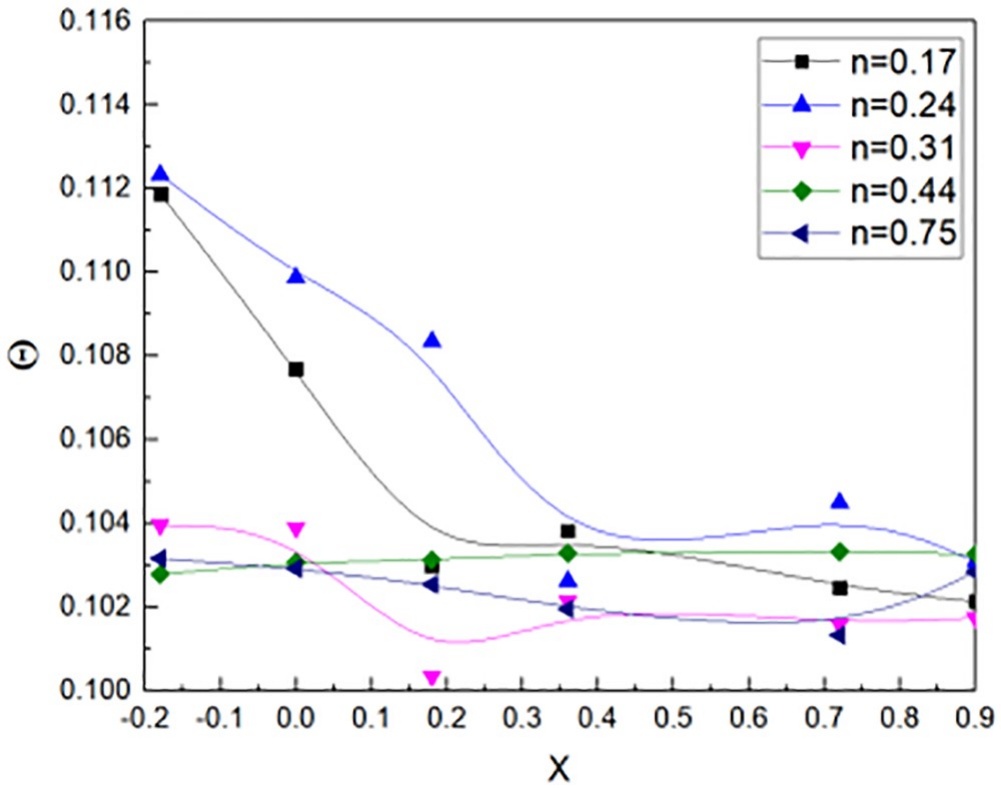
Non-dimensional Temperature by calculation outside window for methanol experimental tests.

The methanol was used as fire source location. According to the Eq (26), the non-dimensional temperature could be calculated. The n was the aspect ratio of the window jet as shown in Eq (22). Fig 9 showed the relation between non-dimensional distance X and the non–dimensional temperature obtained from the methanol experimental results. The window located in the non-dimensional distance X = 0. The n value varied from 0.17 to 0.75, the non-dimensional temperature were more than 0.1. In general, when the n value changed, the dimensionless temperature change was not large.

**Fig 10:**
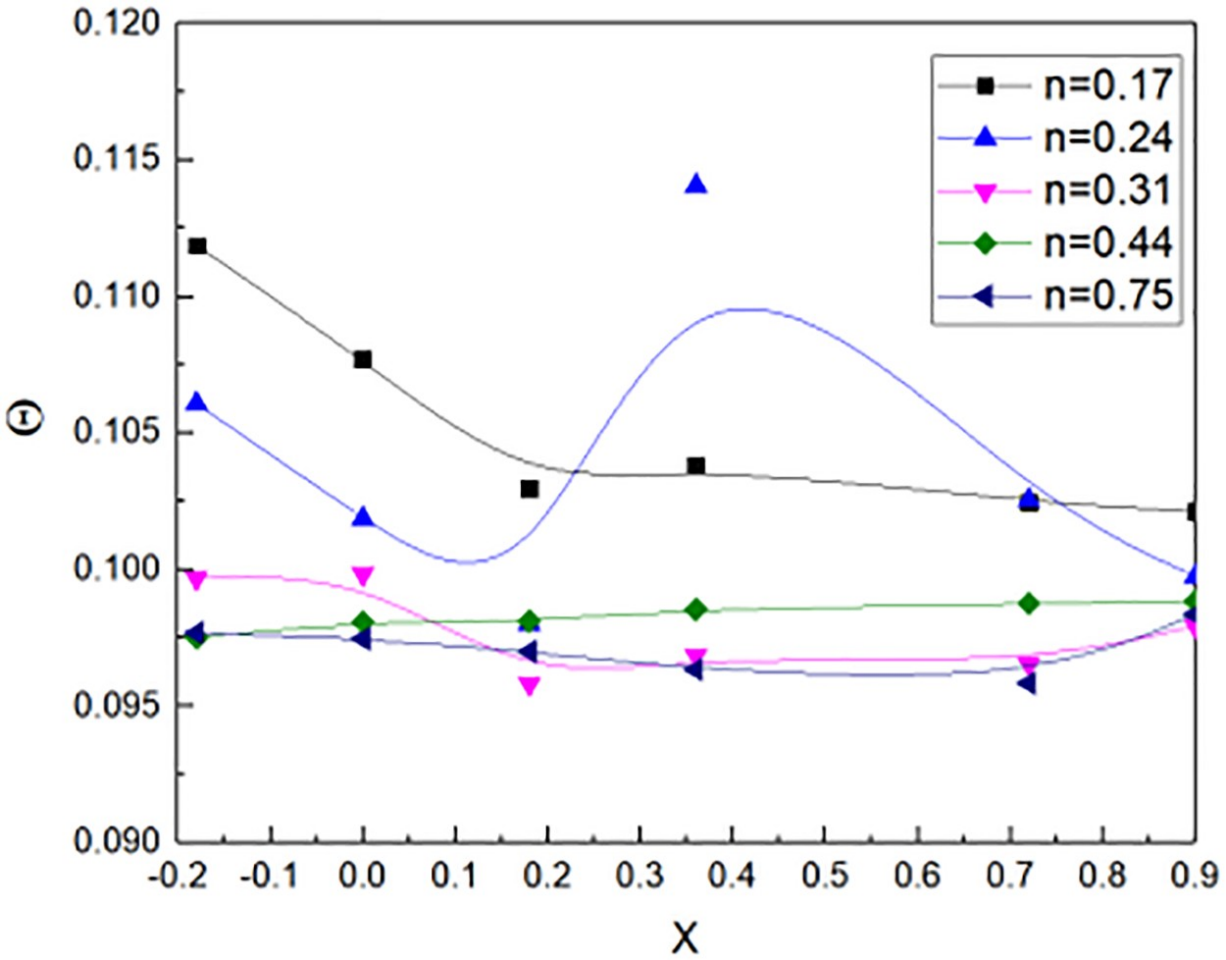
Non-dimensional Temperature outside window for N-heptane experimental tests.

The N-heptane fuel was used as fire source. According to the Eq (26), the non-dimensional temperature could be calculated. The n value is the aspect ratio of the window jet as shown in Eq (22). Fig 10 showed the relation between non-dimensional distance X and the non–dimensional temperature obtained from the N-heptane experimental results. The fire source located in the non-dimensional distance X = 0. The n value varied from 0.17 to 0.75, the non-dimensional temperature were almost 0.1. The non-dimensional temperature fluctuated when the non-dimensional distance changed from the window. In general, when the n value varied, the non-dimensional temperature changed little.

When the non-dimensional distance X is unchangeable. The fuels are methanol and N-heptane. Then, the non-dimensional temperature can be expressed by Eq (31), it can be concluded from the Figs 9 and 10.

$$\Theta (0,0,z^{*})=\{\begin{array}{ll} 0.297{\text{n}}^{3/2}, & \left(\text{n}\leq 3.3\right)\\ 1.782, & \left(\text{n}>3.3\right) \end{array}$$ (31)

### 3.3 Non-dimensional velocity V′

**Fig 11:**
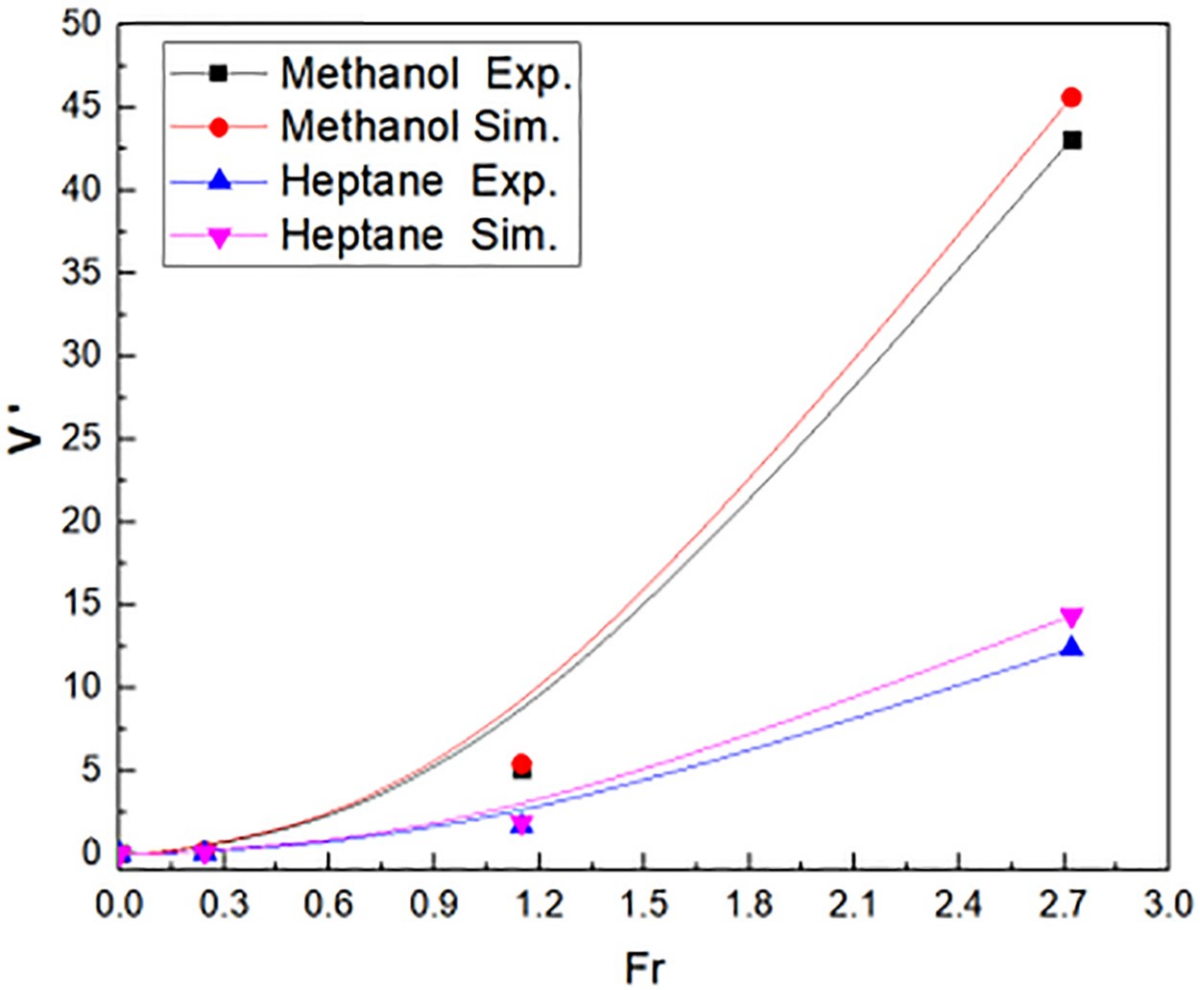
Non-dimensional velocity outside window in reduced scale experiments and simulations.

The non-dimensional velocity outside window in reduced scale experiments and numerical simulations as shown in Fig 11. The fuel of fire source was methanol and N-heptane, respectively. The non-dimensional velocity was calculated by the Eq (30). The non-dimensional velocity increased as the Fr value went up. It was demonstrated that the non-dimensional velocity was calculated by the reduced scale numerical simulations agreed well with the experimental tests.

**Fig 12:**
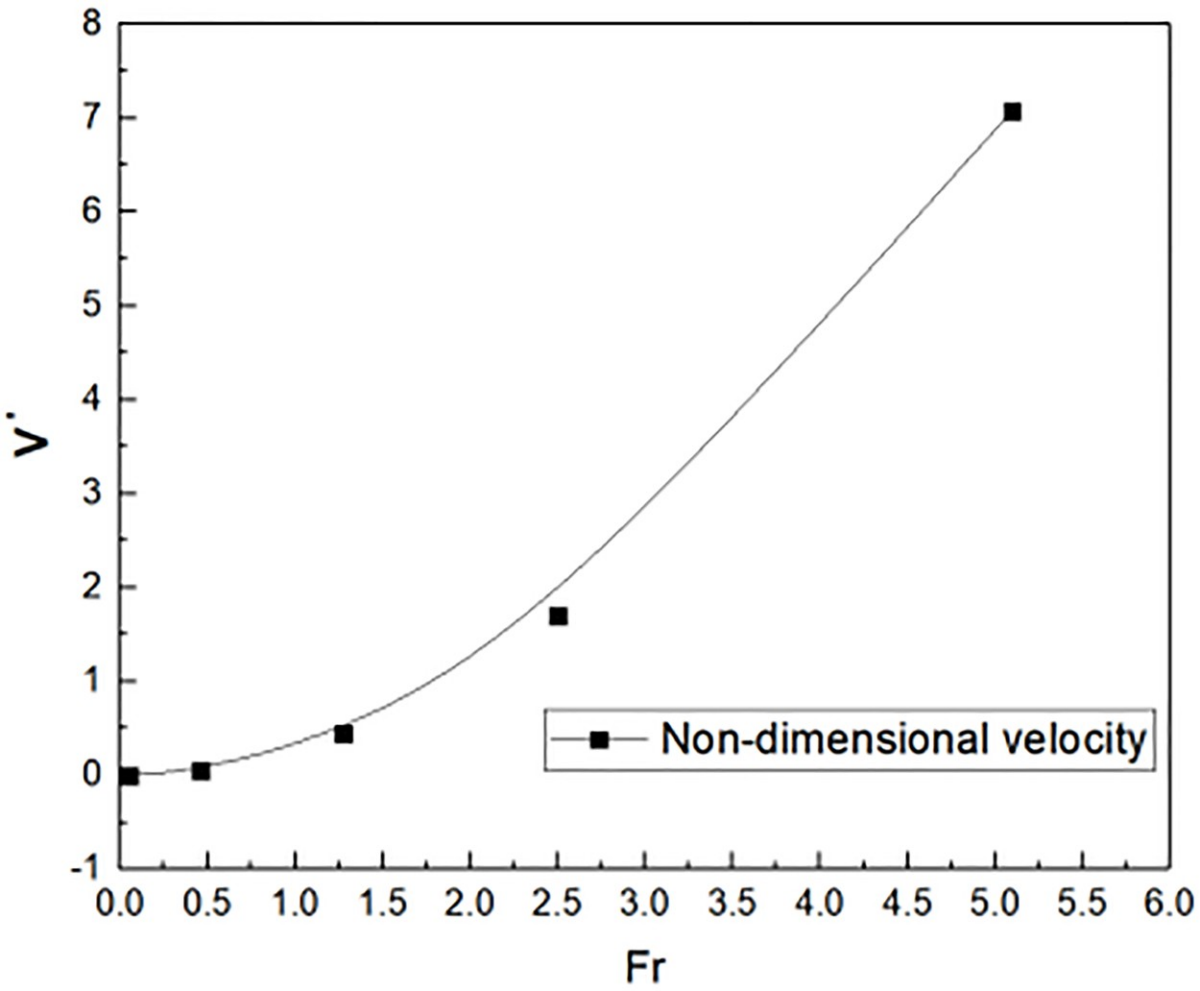
Non-dimensional velocity outside the window distribution in full scale simulations.

The experimental results were plotted in Fig 12, in the definition of the non-dimensional velocity. By the Eq (30), the non-dimensional velocity was obtained in numerical simulations. Namely, the Fr increased, which leaded to the non-dimensional velocity increased gradually. When the Fr was 5.0, the non-dimensional velocity was almost 7. Through the above analysis, the Eq (32) could get the non-dimensional velocity.

$${\text{V}}^{\prime }=0.272{\text{Fr}}^{3/2}$$ (32)

### 3.4. The flame tilt angle θ

**Fig 13:**
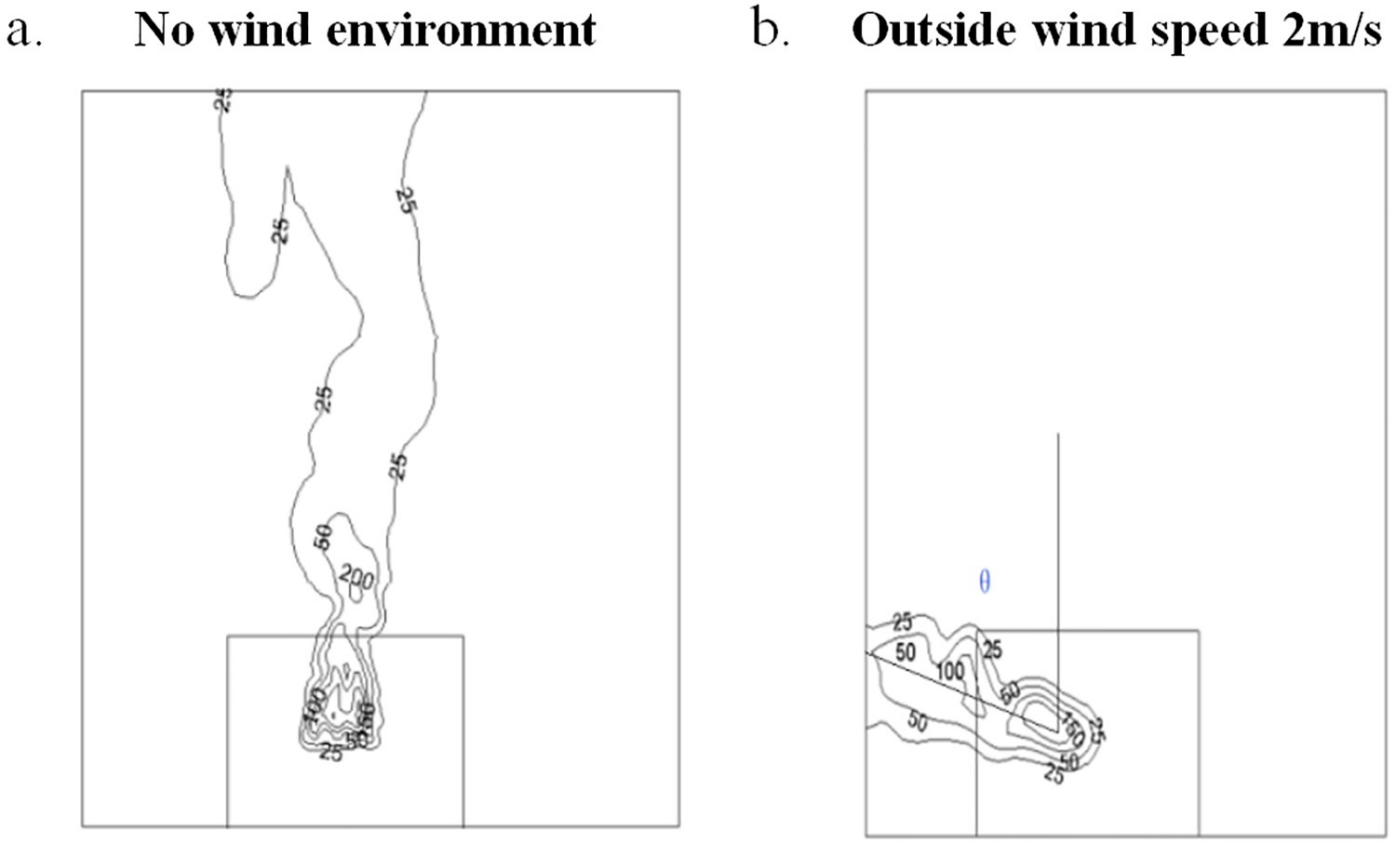
The flame tilt angle when HRR was 6.81kW in numerical simulations. (a) No wind environment. (b) Outside wind speed 2m/s.

**Fig 14:**
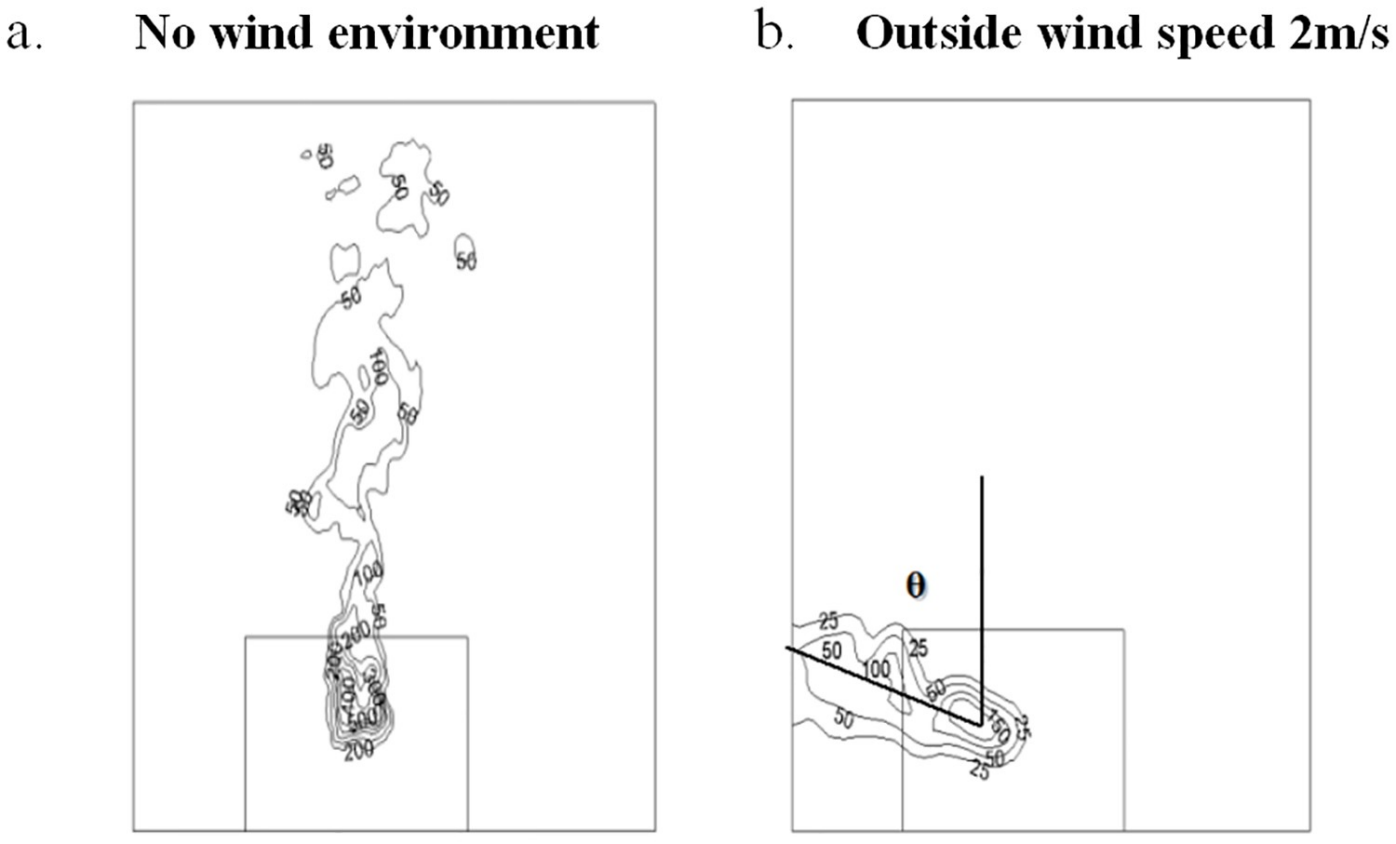
The flame tilt angle when HRR was 9.08kW in numerical simulations. (a) No wind environment. (b) Outside wind speed 2m/s.

**Fig 15:**
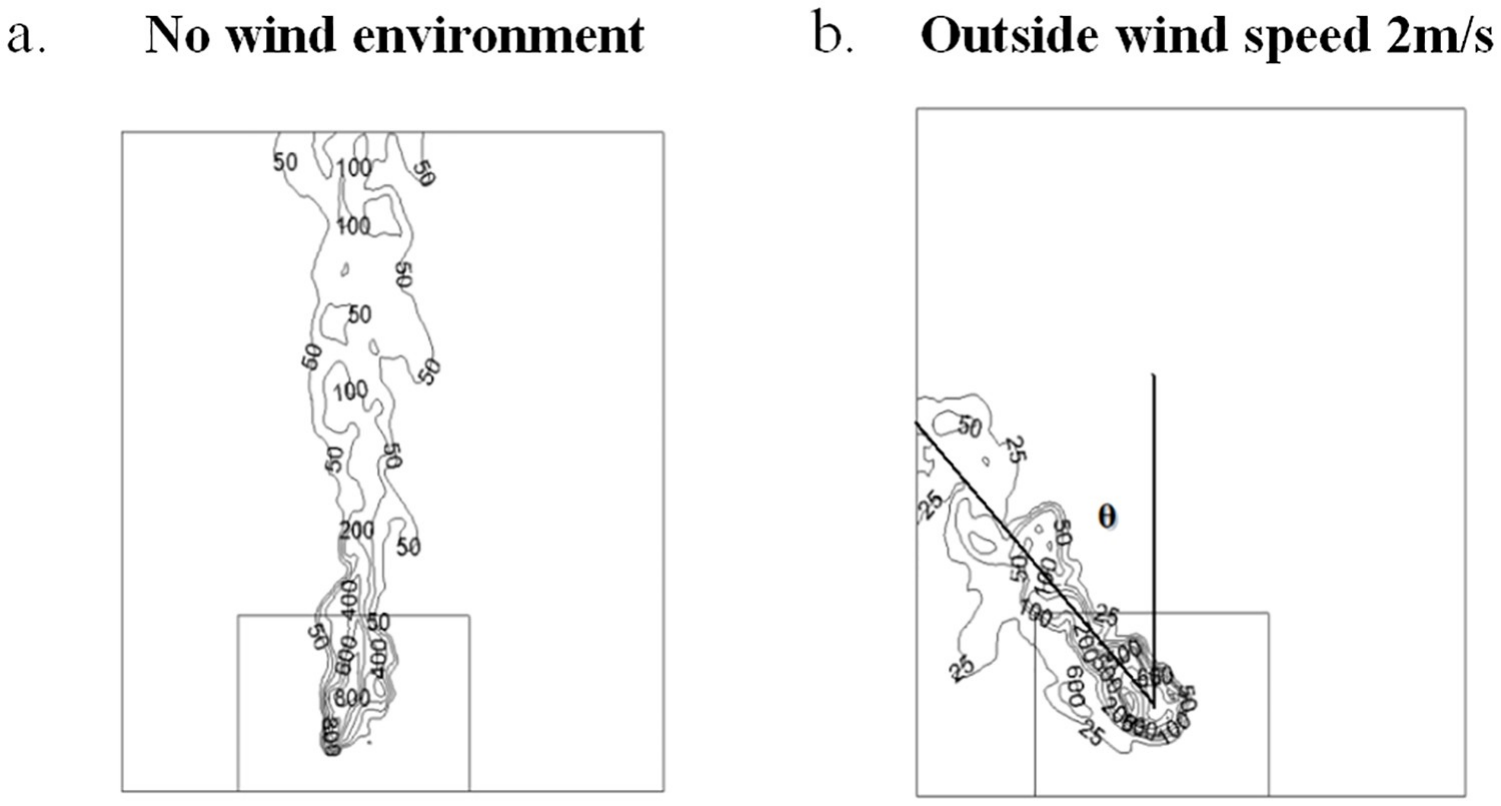
The flame tilt angle when HRR was 17.84kW in numerical simulations. (a) No wind environment. (b) Outside wind speed 2m/s.

In numerical simulations, the flame tilt angle θ could be obtained. In order to study the flame tilt angle varied with the wind speed outside the window, flame temperature distribution at a typical cross section was selected. The typical cross section was 0.1m far away from the building. The flame temperature distribution varied with the distance from the building, however, the flame tilt angle had little effect. Figs 13–15 showed the temperature distribution of 0.1 m cross section at different numerical simulation cases, when the HRRs were 6.81kW, 9.08kW and 17.84kW, respectively. The temperature under these HRRs were 6.81kW, 9.08kW and 17.84kW were compared under the same operating conditions, which the wind speed was 2m/s outside the window. The larger of the HRR fire source, the smaller was the flame tilt angle of fire plume.

**Fig 16:**
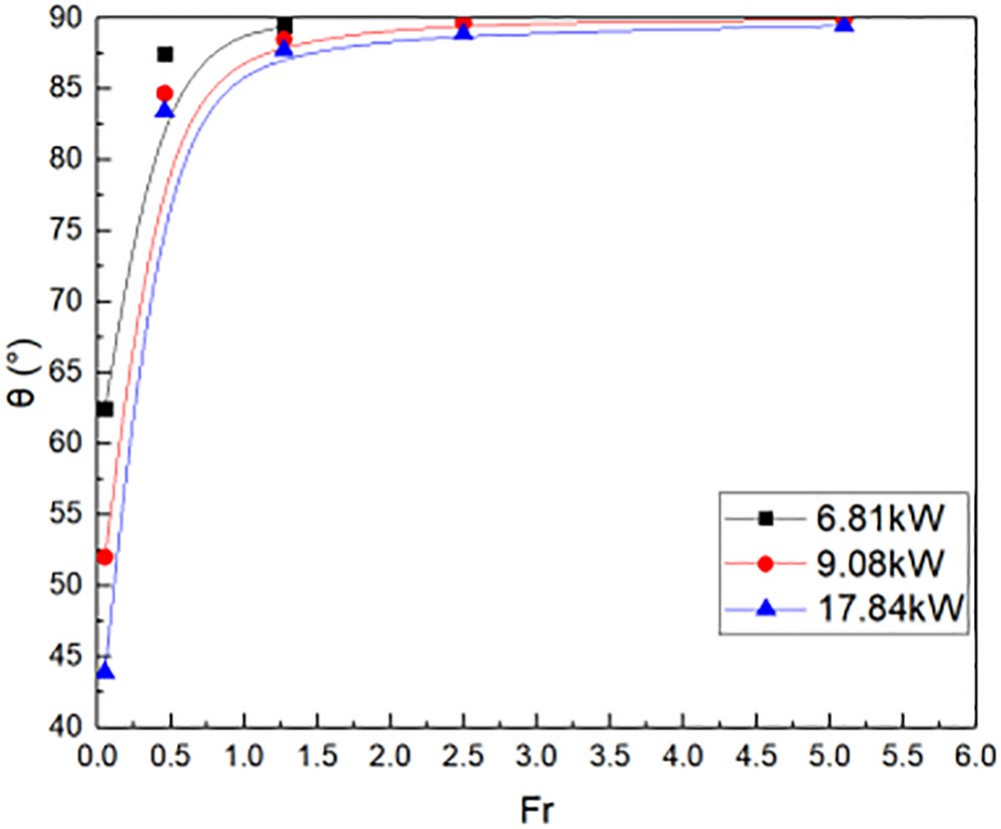
The flame tilt angle outside window in experimental tests.

In experimental tests, the temperature were obtained outside the window. The flame tilt angle θ value was calculated by the Eq (8). The m value was 1.014 by calculation. The maximum HRR were 6.81kW, 9.08kW and 17.84kW in the reduced scale building scene, respectively. It was taken different HRR as fire source, the flame tilt angle were shown in Fig 16 in numerical simulations outside window distributions. There was a clear increase in the θ value with increase in the Fr, for a given HRR. The reason was that the HRR increased, which leaded to the decline of the Fr. Therefore, the flame tilt angle θ decreased as the Fr falls down.

**Fig 17:**
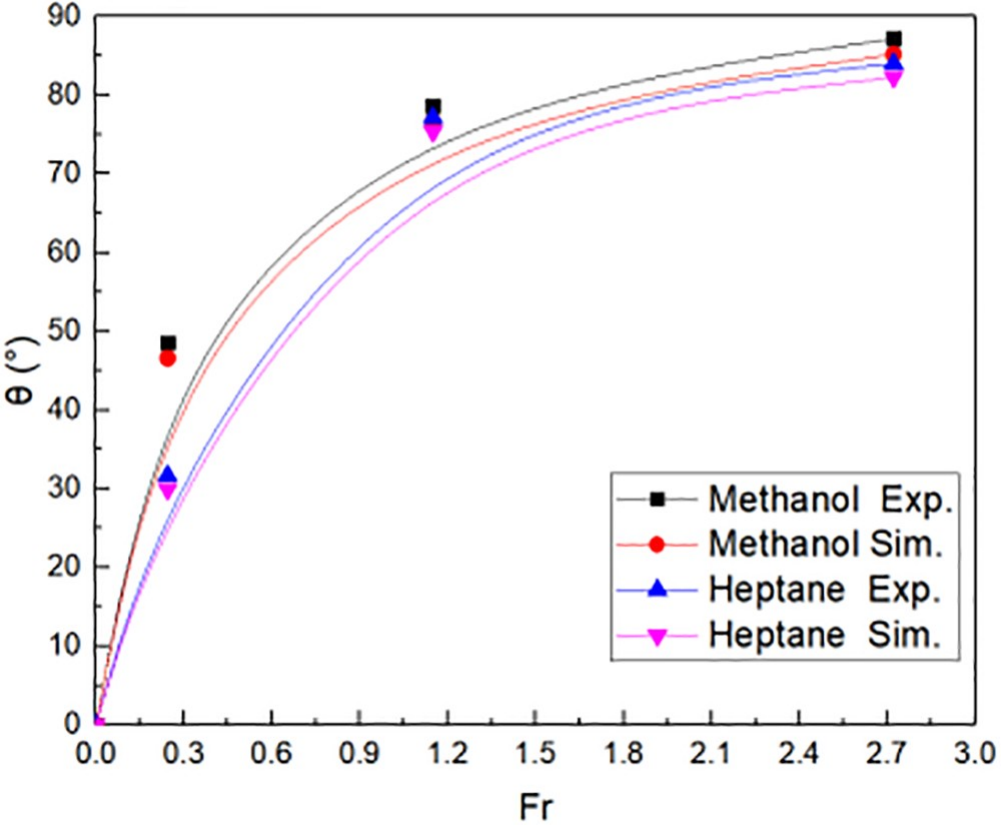
The flame tilt angle outside window in reduced scale experiments and simulations.

Fig 17 illustrated the flame tilt angle θ outside window, which was compared in reduced scale experiments and numerical simulations. The fuel of fire source was methanol and N-heptane, respectively. The flame tilt angle θ was calculated by the Eq (8). The flame tilt angle increased when the Fr varied from 0 to 2.7. The flame tilt angle θ was almost more than 80°. The numerical simulation showed a good agreement with the reduced scale experiments, whatever the fuel of fire source was methanol and N-heptane. Then, the flame tilt angle outside window is shown in Eq (33), which is deduced from Fig 17.

$$\text{tan}\;\theta =-83.74{\text{Fr}}^{-3.43}+84$$ (33)

### 3.5 Comparison with previous study for non-dimensional temperature

**Fig 18:**
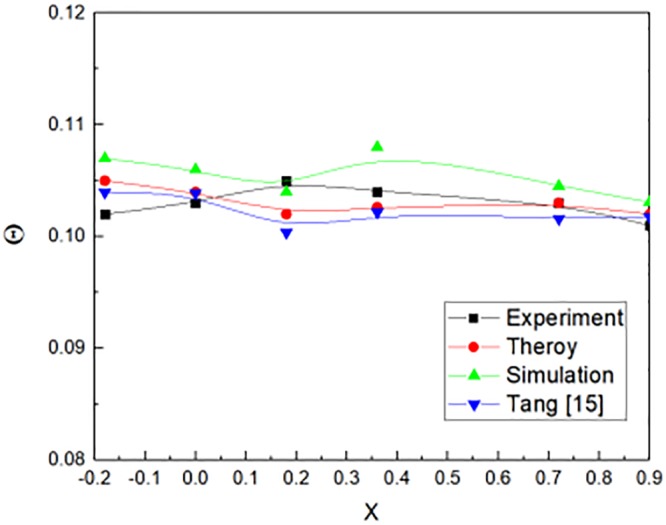
Comparison of this paper with the experimental data from Tang et al. [15].

The paper studied the characteristic of fire plume outside the window. Results were calculated by numerical simulation for predicting the characteristic of fire plume distribution needs to be validated. Tang et al. [15] conducted a scale model experiments to investigate the temperature distribution and flame heights with a 0.8m cubic fire compartment. Fig 18 showed the comparison of non-dimensional temperature in this paper with Tang et.al [15] experimental data. The non-dimensional temperature varied with the non-dimension distance X. It could be verified that the average temperature difference in building facade. The results showed a good agreement with previous studies in the literature. The experimental results obtained the non-dimensional temperature varied with the non-dimension distance X as shown in Eq (34).

$$\Theta =0.103+0.007\text{X-}0{\text{.01X}}^{\text{2}}$$ (34)

## 4. Conclusions

In this study, the combination reduced-scale experimental tests and numerical simulations were employed to study the fire plume characteristics of the building exterior window under lateral blow. It was found that the theoretical equations of the flame tilt angle, the non-dimensional temperature and the non-dimensional velocity outside the window. The fire source fuels were methanol and N-heptane, respectively.

1. The flame tilt angle is independent of the window area and the smoke temperature, but according to the results of reduced scale experimental tests and numerical simulation tests, the flame tilt angle was uniquely determined by the Froude number.
2. The non-dimensional temperature is only affected by the window geometry. The non-dimensional velocity has a great relationship with the Froude number.
3. The numerical simulation results agree well with the results of reduced scale experiments in terms of the flame tilt angle. The results showed that the non-dimensional velocity calculated by the reduced scale numerical simulations agreed well with the experimental tests. Therefore, in order to prevent fire from propagation along the vertical direction of building, the window area and the wind speed around the building should be considered.

Future work should be conducted on the effect of geometry of the window and the impact of the location of the fire in the super high-rise building. This paper mainly studies the fire plume characteristics in building facade window under lateral blow. In the numerical simulation and reduced scale tests, some assumptions were made about the initial boundary conditions, and the influence of the geometry of the window and the location of the fire on the super high-rise building was not considered.

## Abbreviations

${\overset{\cdot }{\text{Q}}}_{\text{inside}}$: Heat release rate inside a combustion chamber
${\text{Q}}_{\text{D}(H)}^{\prime *}$: The ejection height of window jet L_f_
${\text{Q}}_{\text{D}(r_{0})}^{*}$: The non-dimensional heat flow rate of window jet
1/*α*: The inclination of the straight line
A: Fuel area; (m^2^)
B: window width; m
b: Fire overflow width; (m)
b: The width of window plume at the height of Z; (m)
C: Coefficient
C_1_: Window flow coefficient
C_2_: Coefficient
C_p_: Specific heat of air at constant pressure; kJ/(kg·K)
D: Fire source equivalent diameter; m
F_b_: External wind force; (N)
F_f_: Thermal buoyancy; (N)
F_g_: Gravity; (N)
Fr: Froude number
g: Gravity acceleration; (kg/m^3^)
H: The window height; (m)
L_f_: Window plume flame height; (m)
m: Coefficient
m_D_: Mass flow rate of the window jet; kg/s
n: The aspect ratio of the window jet
Q: Heat release rate of fire source; kW
Q*′: The non-dimensional heat release rate of line heat source
Q_D_: The heat ejected along with the window jet
T_0_: Temperature at arbitrary position along the fire plume axis; (°C)
T_0_: Temperature of window fire plume; (°C)
T_a_: Temperature of air; (°C)
T_g_: Window plume temperature; (°C)
u_W_: Lateral blow wind velocity; (m/s)
V′: Non-dimensional velocity
W: Window plume length; (m)
z*: The non-dimensionless height
Z_n_: Neutral plane height at the window;(m)
Θ: Non-dimensional temperature
*θ*: The flame tilt angle
Θ(0,0,z*): The non-dimensionless height z*
*μ*_a_: Aerodynamic viscosity coefficient; (N·s/m^2^)
*ρ*_0_: Window fire plume density; (k/gm^3^)
*ρ*_a_: Ambient air density; (kg/m^3^)
*ρ*_a_: The ambient air density; (k/gm^3^)
*ρ*_g_: Plume flow density; (kg/m^3^)
*ρ*_s_: The window jet density; (k/gm^3^)
